# Development of a dual-expression vector facilitated with selection-free PCR recombination cloning strategy

**DOI:** 10.1186/s13568-017-0386-1

**Published:** 2017-05-16

**Authors:** Liting Cao, Yancheng Zhou, Lin Huang, Shiqi Dong, Yue Ma

**Affiliations:** Rongchang Campus, Southwest University, Rongchang, Chongqing, 402460 China

**Keywords:** PCR cloning, Prokaryotic expression, Eukaryotic expression, Lysis gene E, Positive selection

## Abstract

The conventional procedure for the construction of recombinant expression vector of a target gene includes PCR cloning and restriction enzyme mediated subcloning, which is time-consuming and sometimes troublesome because of the inefficiency of ligation. A variety of ligase-independent PCR cloning strategies have been developed, but they either involve complicated PCR procedures or need other DNA modifying enzymes. In this study, we report the design, and construction of an omnipotent expression vector pOmni, with which a target gene can be easily cloned through innovative selection-free PCR recombination cloning strategy with only one pair of primer and two times of PCR in one work day, without using any restriction enzymes, ligase and other DNA modifying enzymes. Furthermore, the target gene cloned in pOmni is ready to be high-efficiently expressed in either *Escherichia coli* cells or eukaryotic cells because of the elaborate design of compatible T7 promoter and CMV promoter expression elements in the vector. The cloning capability and reliability of selection-free PCR recombination cloning with pOmni were validated through cloning of 6 DNA fragments with length from 315 to 4557 bp, and the dual-expression function of the vector was verified through the cloning and expression of EGFP in *E. coli* BL21 and HeLa cells. pOmni developed in our study provides a powerful tool for gene cloning and expression, and is of special value for researches in which both prokaryotic and eukaryotic expression of a target gene are necessary.

## Introduction

Expression of a target gene in prokaryotic cell or/and eukaryotic cell has become a common research goal or a basic experimental procedure for profound researches of genes of interest since Herbert Boyer and Stanley N. Cohen initiated the genetic engineering era. PCR has been indispensible for gene cloning because it can provide target gene fragments in vitro in large amount and short time without the limitation of restriction sites. As a result, the mainstream strategy for the construction of recombinant expression vector includes two cloning steps: PCR cloning (cloning of PCR amplified target gene into cloning vector) and restriction enzyme mediated subcloning of target gene from cloning vector into expression vector. Direct cloning of PCR amplified target gene into expression vector via double-restriction of PCR products is optional, but the uncertainty of the accuracy of artificially synthesized restriction site in PCR primers makes trouble-shooting difficult if the cloning experiment fails.

T-A cloning (Marchuk et al. 1991) is the most commonly used PCR cloning strategy because many cheap commercial T-A cloning kits are available. Blunt-end PCR cloning kit is also available, but it has in fact the same drawback like T-A cloning because both of them are ligase-dependent. The ligation efficiency of blunt-ends and 3′mononucleotide overhang is usually unsatisfied, especially when inserts are comparatively long. Restriction enzyme mediated subcloning is more time-and-money consuming because two times of double-restriction and gel purification are needed. Almost 1 week is necessary even for an experienced researcher to construct recombinant expression vector of a target gene by means of above cloning strategy.

Many ligase-independent PCR cloning strategies have been developed in order to overcome the inefficiency of ligase dependent PCR cloning, such as ligation independent cloning (Aslanidis and Dejong 1990; Rashtchian et al. 1992; Li and Evans 1997; Weeks et al. 2007), recombinase-mediated cloning (Walhout et al. 2000; Cheo et al. 2004), and PCR-mediated cloning (Shuldiner et al. 1990; Tillett and Neilan 1999; Ent and Löwe 2006; Zuo and Rabie 2009; Bryksin and Matsumura 2010).

Among PCR-mediated cloning strategies, the so-called restriction-free cloning (Ent and Löwe 2006) and overlap extension PCR cloning (Bryksin and Matsumura 2010) utilizes identical technical mechanism and can be used to clone PCR amplified target gene directly into expression vector. In this cloning strategy, PCR amplified target gene is inserted into plasmid vector by means of a lineal recombination PCR using circular vector as template and target gene PCR products as primers, which contains two primer-introduced terminal sequences homologous to correspondent sequences in the vector. ‘PCR recombination cloning (PCRRC)’ would be a better terminology for this cloning strategy because it is a simulation of natural double-cross homologous recombination, in which the vector sequence between the two homologous region is replaced by the cloned target gene and results in nicked circular recombinant plasmid. Although the PCRRC strategy in these two reports is more straightforward, efficient and reliable compared with other PCR-mediated cloning strategies, a restriction enzyme (*Dpn*I) digestion step is still necessary to eliminate template vector as selection for recombinant plasmids.

In order to overcome above mentioned inconvenience of constructing recombinant expression vector of a target gene, we elaborately designed and constructed an omnipotent expression vector designated as pOmni that has two advantageous technical features. First, pOmni is a vector facilitated with selection-free PCRRC function, which makes *Dpn*I digestion in reported PCRRC strategy (Ent and Löwe 2006; Bryksin and Matsumura 2010) unnecessary. This technical innovation of pOmni makes it possible to construct a recombinant expression vector in 8 h by starting with the PCR amplification of the target gene. Second, pOmni is a pro/eukaryotic dual-expression vector containing compatible eukaryotic CMV expression elements and prokaryotic T7 expression elements, which would greatly benefit researches in which both prokaryotic and eukaryotic expression of a target gene are necessary.

## Materials and methods

### Bacterial strains, plasmids, and cultivation conditions

All bacterial strains and plasmids used in this study are listed in Table 1. All *E. coli* strains were cultured in Luria–Bertani (LB) medium (Sambrook and Russell 2001) or on LB agar plates containing 100 mg/l ampicillin except that for competent cell preparation.

**Table 1:** Strains and plasmids used in this study

Strain or plasmid	Description	Reference
Strains		
*E. coli* DH5α	F- Phi80lacZDeltaM15 Delta(lacZYA-argF) U169 recA1 endA1 hsdR17(rk−, mk +) phoA supE44 thi-1 gyrA96 relA1 tonA	Invitrogen
*E. coli* D1210HP	F- recA56 thi-1 leu-6 proA2 hsdS20 (rB-mB-) lacY1 galK2 ara-14 mtl-1 xyl-5 lacIq lacY1 endA rpsL20 (Lambdaxis-kil-cI857)	Merck
Plasmids		
pcDNA 3.1(+)	Vector for high-level expression in mammalian cells	Invitrogen
pElysM	Highly efficient host-killing vector	Ma et al. (2009)
pC3.1	Derivative of pcDNA 3.1(+), lacking SV40 origin and neomycin selection gene	This study
pC3.2	Derivative of pC3.1, containing Lac operator, ribosome biding site (RBS), Kozak sequence and 6His tag	This study
pC3.3	Derivative of pC3.2, containing T7 terminator	This study
pOmni	Derivative of pC3.3, containing lysis gene E expression cassette	This study
pIRES2-EGFP	EGFP reporter expression vector	Clontech

### DNA synthesis and manipulation

All mutational primers and regular primers were designed with Primer Premier 5.0, and PAGE-purification grade primers were synthesized by Sangon Biotech Co. Ltd. (Shanghai, China). All PCR products were visualized through agarose-gel electrophoresis if necessary. Plasmid DNA was isolated using the ‘E.N.Z.A Plasmid Mini Kit’ (Omega Bio-Tek, USA). PCR products were gel-purified using the “E.N.Z.A Gel Extraction Kit (Omega Bio-Tek, USA)” when needed. Transformation of *Escherichia coli* in all cloning experiment was conducted according to the standard chemical transformation protocol (Sambrook and Russell 2001).

### Vector design and construction

pOmni was designed and constructed based on commonly used eukaryotic expression vector pcDNA 3.1(+) through a series of PCR-mediated mutation.

Intermediate vector pC3.1 was firstly constructed through mutation of pcDNA 3.1(+) with primer set 5′CTCGGTCGTTCGGCTGCG3′ and 5′GGCGCGTGGGGATACCC3′, by which the SV40 origin and neomycin selection gene region was deleted. MutanBest Kit (Takara Bio, Dalian, China) was used for PCR-mediated mutation according to manufacturer’s manual. In brief, the PCR products amplified with above primer set and pcDNA 3.1(+) as template were gel purified, phosphorylated, self-ligated, and transformed into DH5α.

Intermediate vector pC3.2 was constructed based on pC3.1 by means of the same mutation protocol mentioned above with primer set 5′GCTAGCGGAATGTAGCGGATAACAATTCCCCTCTAGAAATAATTTTGTTTAACTTTAAG*AAGGA*GATATGC AGC AGC **CAC CAT CAT CAC CAC CAC** GTTTAAACTTA AGCTTGGTACCGAGC3′ and 5′ CAGCTTGGGTCTCCCTATAGTGAGT3′, by which the *Lac* operator (shaded sequence), a ribosome biding site (RBS, italic sequence), a Kozak sequence (framed sequence) (Kozak 1987) and a 6His tag (bold sequence) were introduced between T7 promoter and BGH Poly(A) site.

Intermediate vector pC3.3 was constructed based on pC3.2 by means of the same mutation protocol mentioned above with primer set 5′CTAGCATAACCCCTTGGGGCCTCTAAACGGGTCTTGAGGGGTTTTTTGTTCGAAGGCGGTAATACGGTTATCCACAG 3′ and 5′ AGCCATAGAGCCCACCGCA 3′, by which T7 terminator (underlined sequence) for prokaryotic expression was introduced into a site downstream BGH Poly(A) site.

The destination vector pOmni was constructed based on pC3.3 by means of reported PCRRC strategy (Ent and Löwe 2006; Bryksin and Matsumura 2010) with little modification. Lysis gene E expression cassette without cI857 supressor gene was amplified with Q5 high-fidelity DNA Polymerase (New England Biolabs, USA) from the host-killing vector pElysM (Ma et al. 2009) with primer set 5′GGGCAGCAGCCACCATCATCACCACCACACCTACCAAACAATGCCCC3′ and 5′GCAACTAGAAGGCACAGTCGAGGCACAGAAGCTTGGCTGCAGTAC3′. The 5′ termini of the forward and reverse primer contain an extra 22nt sequence (underlined sequence) respectively homologous to 6His tag region and BGH reverse sequencing primer biding region in pC3.3. A 20 μl recombination PCR (95 °C 30 s, 60 °C 45 s, 72 °C 3 min, 20 cycles) was conducted using 250 ng gel purified PCR products of lysis gene E expression cassette as primer and 5 ng pC3.3 as template. 5 μl recombination PCR product was directly used to transform *E. coli* D1210HP that provides temperature-dependent transregulation of the expression of lysis gene E. All transformants were cultured on Amp-LB plate overnight at 28 °C. 10 colonies were randomly picked and cultured in 5 ml Amp-LB at 28 °C for 1 h and then half of the cultures was aliquoted into a new sterilized tube and cultured at 42 °C overnight. The clone that grew at 28 °C but not at 42 °C is the positive clone harboring pOmni (Genbank Accession number: KY608793).

### Establishment of selection-free PCRRC strategy with pOmni

DNA fragments with length of 315, 1041, 2504, 3486 and 4557 bp were amplified from irrelevant DNA templates with Q5 high-fidelity DNA polymerase according to regular PCR procedure, respectively. All forward primers and reverse primers contain 5′CAGCCACCATCATCACCACCAC3′ and 5′TGCACGTAATTTTTGACGCACG3′ at the 5′ termini respectively, which are homologous to correspondent sequences flanking the lysis gene E expression cassette in pOmni (Fig. 2).

Individual PCR product was cloned into pOmni through a 20 μl recombination PCR using Q5 high-fidelity DNA polymerase, which contained 5 ng pOmni as template and 100–200 folds (molar ratio) gel-purified PCR product as primer. All recombination PCRs were conducted with the same thermal cycling parameter (95 °C 30 s, 60 °C 45 s, 72 °C 3 min). 5 μl products of recombination PCR was directly used to transform DH5α competent cells, and all transformation product was cultured on Amp-LB plate at 37 °C overnight. The quantity of clones of individual cloning was count, and then 10 clones from individual cloning were randomly picked and regular colony PCR with correspondent primer set was conducted to confirm positive clones.

### Cloning and expression of EGFP

Complete coding sequence of EGFP gene was amplified from pIRES2-EGFP (Clontech, USA) and cloned into pOmni according to the same selection-free PCRRC protocol with primer set 5′CAGCCACCATCATCACCACCACATGGCCA CAACCATGGTGAG3′ and 5′TGCACGTAATTTTTGACGCACGTTACTTGTACAGCTCGTCCAT3′. The only modification of the protocol is that BL21 competent cell was used to transform the recombination PCR product and all transformation products were cultured on Amp-LB plate containing 2 mM IPTG. The expression of EGFP in BL21 was checked and photographed under long-wave ultraviolet light.

A randomly picked pOmni-EGFP clone was cultured in 5 ml Amp-LB overnight, and the recombinant vector was extracted. 500 ng pOmni-EGFP was used to transfect HeLa cells with TransFectin (Bio-Rad, USA) according to the manufacturer’s instruction. The expression of EGFP in transfected HeLa cells was checked and photographed by confocal laser scanning microscope (Zeiss LSM 780).

## Results

### Vector design and construction

**Fig. 1:**
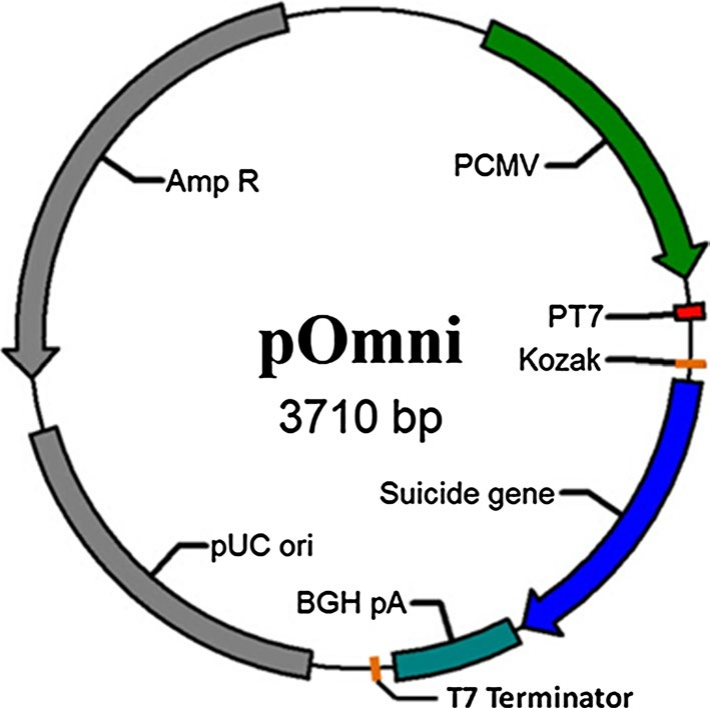
Physical map of pOmni. Suicide gene (expression cassette of lysis gene E of bacteriophage Phi-X174) is to be replaced by cloned gene in PCRRC strategy so as to serve as positive selection gene for recombinant vector

**Fig. 2:**
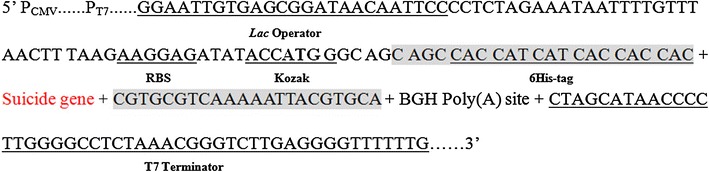
Detailed sequence information of designed functional elements in pOmni. *Lac* operator, ribosome biding site (RBS), Kozak sequence and 6His tag were introduced between T7 promoter and BGH Poly(A) site. PCR amplified target gene products containing two primer-introduced terminal sequences (*shaded sequences*) can be directionally and seamlessly inserted into correspondent position and replace the suicide gene through recombination PCR. The designed Kozak sequence and its proper position related to RBS make it possible to express cloned target gene in both *E. coli* and eukaryotic cells

The designed vector pOmni (Fig. 1) was constructed on the basis of pcDNA 3.1(+) through four times of PCR-mediated mutation, in which one is deletion and three are insertions. Through two times of insertion mutation, the *Lac* operator and ribosome biding site (RBS) was introduced downstream T7 promoter originally used for sequencing in pcDNA 3.1, and T7 terminator was introduced downstream BGH Poly(A) site so as to realize the controlled expression of cloned gene in *E. coli*. A Kozak sequence ‘ACC**ATG**G’ was introduced at the position 5 bp downstream RBS, which makes the starting codon in the Kozak sequence can be shared in both eukaryotic and prokaryotic expression. A 6His tag was also introduced adjacent to the Kozak sequence, and as a result, the cloned target gene can be expressed as a 6His tag fusion protein (Fig. 2).

In order to realize a selection-free PCRRC function in the designed vector, the expression cassette of lysis gene E of bacteriophage Phi-X174 as suicide gene was introduced into pOmni between the 6His tag region and BGH Poly(A) site (Fig. 2). As a result, PCR products containing two primer-introduced terminal sequences (5′CAGCCACCATCATCACCACCAC3′ and 5′TGCACGTAATTTTTGACGCACG3′) that are homologous to the region flanking lysis gene E expression cassette can be inserted into pOmni and substitute lysis gene E expression cassette by means of PCRRC strategy, and the transformants of recombinant vector can be selected automatically.

### Selection-free PCRRC strategy with pOmni

In order to validate the reliability, capability and efficiency of selection-free PCRRC strategy based on pOmni, five amplified DNA fragments varying in length from 315 to 4557 bp were cloned by means of selection-free PCRRC strategy. The results showed that all the 5 PCR products were successfully cloned, which proved the reliability and capability of pOmni-based PCRRC strategy. Colony PCR of 10 randomly picked clones of each cloning test showed 100% positive clone rate, which confirmed the efficiency of selection-free function of pOmni. The cloning efficiency (number of clones formed per transformation) of selection-free PCRRC with pOmni was negatively correlated to the length of cloned fragments (Table 2), which is in agreement with the result in another PCRRC reported (Bryksin and Matsumura 2010).

**Table 2:** Cloning capability and efficiency of selection-free PCRRC with pOmni

Length of cloned fragments (bp)	Number of clones	Positive clone rate (positive/negative)
315	473	10/0
1041	312	10/0
2504	138	10/0
3486	74	10/0
4557	36	10/0

### Pro/eukaryotic dual-expression capability of pOmni

**Fig. 3:**
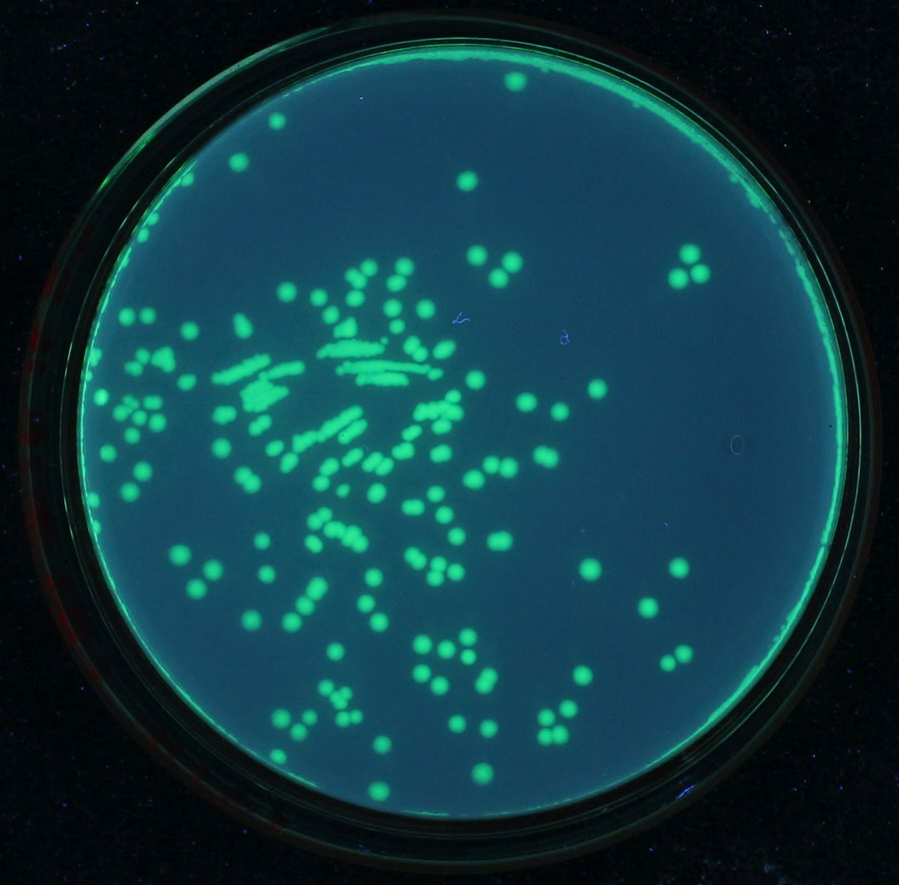
Expression of EGFP cloned in pOmni in *E. coli* BL21. EGFP gene was cloned into pOmni by means of selection-free PCR recombination cloning. All BL21 transformants showed *strong green* florescence under long-wave ultraviolet light

**Fig. 4:**
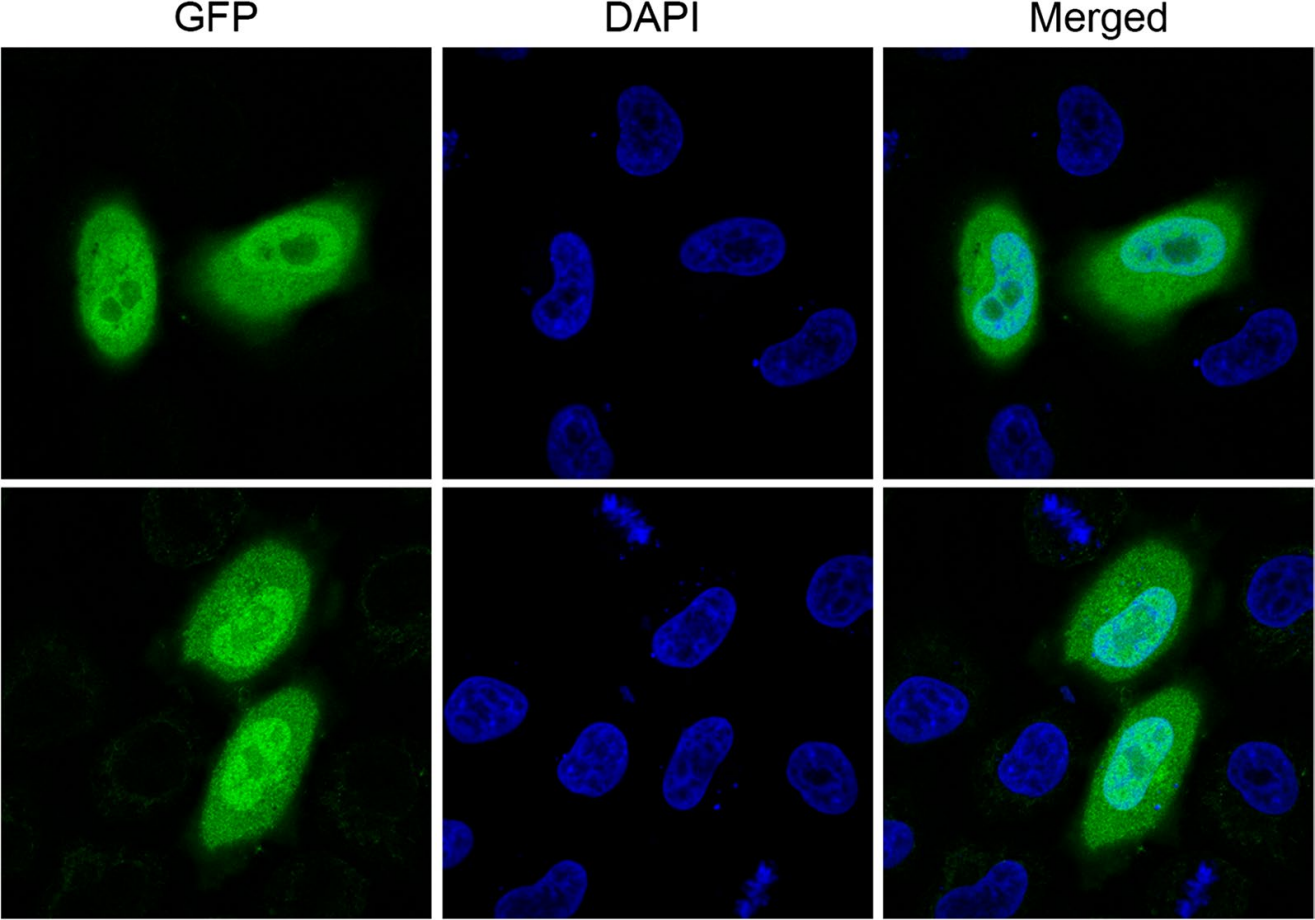
Expression of EGFP cloned in pOmni in HeLa cell. Confocal laser scanning microscopy showed that EGFP cloned in pOmni was strongly expressed in transfected HeLa cells

In order to verify the dual-expression capability of pOmni, EGFP gene was cloned into pOmni by means of selection-free PCRRC strategy. The result showed that about 150 clones grew after transformation of BL21 with 5 μl untreated recombination PCR products. All transformant clones showed strong green florescence under long-wave ultraviolet light (Fig. 3), which indicates that a target gene cloned with pOmni via PCRRC strategy can be correctly and high-efficiently expressed in *E. coli*. The 100% positive clone rate (150/150) in EGFP cloning experiment reconfirmed the selection-free function of pOmni-based PCRRC strategy. Clear green fluorescence all over the cytoplasm of HeLa cells transfected with pOmni-EGFP was showed by confocal laser scanning microscopy (Fig. 4). It indicates that a target gene cloned in pOmni via PCRRC strategy can also be high-efficiently expressed in eukaryotic cells.

## Discussion

The reported PCRRC (Ent and Löwe 2006; Bryksin and Matsumura 2010) has been the most simple and efficient strategy of all ligase-independent PCR cloning strategies because the other strategies either involve complicated PCR procedures (Shuldiner et al. 1990; Tillett and Neilan 1999; Zuo and Rabie 2009) or need other DNA modifying enzymes (Aslanidis and Dejong 1990; Rashtchian et al. 1992; Li and Evans 1997; Weeks et al. 2007). However, they are not truly restriction enzyme independent because *Dpn*I digestion as positive selection for recombinant vector is indispensible in the reported PCRRC strategy. This is because the transformation efficiency of the supercoiled vector as template in recombination PCR is more efficient than the nicked recombinant plasmid formed in recombination PCR, which means unacceptable background (false positive) clones would take place without *Dpn*I digestion.

The important innovation of the designed vector is the utilization of the lysis gene E of bacteriophage Phi-X174 as positive-selection gene in pOmni (Fig. 2), which makes *Dpn*I digestion step longer necessary in reported PCRRC and results in decreasing the time and money cost of cloning. To the author’s knowledge, no suicide gene-dependent positive selection mechanism has been used in reported ligase-independent PCR cloning strategies. The expression product of lysis gene E is lethal to *E. coli* (Henrich et al. 1982; Witte et al. 1990, 1992) and some other Gram-negative bacteria (Ronchel et al. 1998; Katinger et al. 1999; Marchart et al. 2003; Wei et al. 2011) through tunnel formation on cell membrane (Witte et al. 1990, 1992). Lysis gene E is an ideal positive-selection gene for cloning vector because the lethal efficiency of lysis gene E expression product to commonly-used *E. coli* cloning strains is as high as 99.999% (Ma et al. 2009). Another technical advancement in pOmni compared with the reported lysis gene E-dependent cloning vector (Ma et al. 2009) is the exclusion of suppressor gene cI857 from the lysis gene E expression cassette, which makes culturing of transformants at 42 °C as positive selection measure unnecessary.

These elaborate designs make pOmni a selection-free PCRRC vector having following advantages. (1) The selection-free PCRRC strategy with pOmni is the simplest and fastest one of all reported ligase-independent PCR cloning strategies, by which a target gene can be cloned in one work day with only one pair of primer and two times of PCR by starting with the amplification of the target gene, without using any tool enzymes except high-fidelity DNA polymerase. (2) The selection-free PCRRC strategy with pOmni is highly efficient and reliable. 6 DNA fragments with length from 315 to 4557 bp were successfully cloned in the validation experiments, and the 100% positive clone rate of 10 randomly-picked clones in each cloning experiment was verified by means of colony PCR. (3) The selection-free PCRRC strategy with pOmni is of special value for cloning of large PCR fragments. To our knowledge, cloning of PCR fragments larger than 2500 bp with conventional T-A cloning strategy is usually difficult, but all 3 large DNA fragments (2504, 3486 and 4557 bp) were easily cloned in our validation experiments.

Other two technical details of the design consummate pOmni as a PCRRC vector. First, the 6His tag sequence in the 22 bp upstream homologous recombination region (5′CAGCCACCATCATCACCACCAC3′, 6His tag underlined) was redesigned (compared with 6His tag sequence in the famous pET serial vectors) to ensure the efficiency of recombination PCR. As a result, all validation experiments cloning DNA fragments with different length succeeded without any optimization of the recombination PCR parameters. Second, the deletion of episomal SV40 origin and neomycin selection gene region of pcDNA 3.1 diminished the size of the designed vector by more than 2 kb, which would greatly increase the capability of the designed vector to clone large PCR products.

Pro/eukaryotic dual-expression vector is of special application value for researches in which both prokaryotic and eukaryotic expression of a target gene are necessary. In DNA vaccine researches, for example, prokaryotic expression of the candidate gene is usually necessary to prepare antiserum for verification of the antigenicity of the candidate gene or to prepare antigen for monitoring the efficiency of the DNA vaccine (eukaryotic expression vector of the target gene). The introduction of prokaryotic expression elements and the precise design of the compatible RBS and Kozak sequence endow pOmni with pro/eukaryotic dual-expression capability, which was validated by cloning and expression of reporter gene EGFP in *E. coli* and HeLa cells in this study. pOmni has two technical advantages compared with the only pro/eukaryotic dual-expression vector pDual GC (Agilent, USA) that we know. (1) pOmni is much smaller in size (3.7 vs. 6.6 kb) that means higher cloning capability for large DNA fragments. (2) pOmni has selection-free PCRRC function as discussed above, but with pDual GC, a target gene has to be cloned by means of traditional and troublesome restriction-mediated cloning method.

## Abbreviations

PCRRC: PCR recombination cloning
PCR: polymerase chain reaction
CMV: cytomegalovirus
